# Synthesis, Antibacterial, and Antioxidant Evaluation of Novel Series of Condensed Thiazoloquinazoline with Pyrido, Pyrano, and Benzol Moieties as Five- and Six-Membered Heterocycle Derivatives

**DOI:** 10.3390/molecules26020357

**Published:** 2021-01-12

**Authors:** Ebraheem Abdu Musad Saleh, Abdullah Mohammed AL Dawsari, Kakul Husain, Ismail Hassan Kutty, K.M.Lokanatha Rai

**Affiliations:** 1 Department of Chemistry, College of Arts and Science, Prince Sattam Bin Abdulaziz University, Wadi Al-Dawasir 11991, Saudi Arabia; abdullah.aldawsari@psau.edu.sa (A.M.A.D.); k.firoz@psau.edu.sa (K.H.); ismailpanavally@gmail.com (I.H.K.); 2 Department of Studies in Chemistry, University of Mysore, Manasagangotri, Mysore 570 006, India; kmlrai@yahoo.com

**Keywords:** α,α dioxoketen dithioacetal, pyrido, pyrano, benzo, thiazolo, quinazoline, antibacterial and antioxidant

## Abstract

A novel synthesis of thiazolo[2,3-b]quinazolines **4**(**a**–**e**), pyrido[2′,3′:4,5]thiazolo[2,3-b]quinazolines {**5**(**a**–**e**), **6**(**a**–**e**), and **7**(**a**–**e**)}, pyrano[2′,3′:4,5]thiazolo[2,3-b]quinazolines **8**(**a**–**e**), and benzo[4,5]thiazolo[2,3-b]quinazoloine**9**(**a**–**e**) derivatives starting from 2-(Bis-methylsulfanyl-methylene)-5,5-dimethyl-cyclohexane-1,3-dione **2** as efficient α,α dioxoketen dithioacetal is reported and the synthetic approaches of these types of compounds will provide an innovative molecular framework to the designing of new active heterocyclic compounds. In our study, we also present optimization of the synthetic method along with a biological evaluation of these newly synthesized compounds as antioxidants and antibacterial agents against the bacterial strains, like *S. aureus*, *E. coli,* and *P. aeruginosa*. Among all the evaluated compounds, it was found that some showed significant antioxidant activity at 10 μg/mL while the others exhibited better antibacterial activity at 100 μg/mL. The results of this study showed that compound **6**(**c**) possessed remarkable antibacterial activity, whereas compound **9**(**c**) exhibited the highest efficacy as an antioxidant. The structures of the new synthetic compounds were elucidated by elemental analysis, IR, ^1^H-NMR, and ^13^C-NMR.

## 1. Introduction

Considerable research efforts have been devoted to synthesizing thiazole, quinazoline derivatives, and their moieties because of their widespread applications of biological merit [1,2,3,4]. Thiazole and quinazoline exist in condensed fused systems such as thiazolo[3,2-*a*]quinazoline, thiazolo[4,3-*b*]quinazoline, thiazolo[2,3-*b*]quinazoline, 8*H*-thiazolo[5,4-*f*]quinazolin-9-ones, thiazolo[4,5-*h*]quinazolin, and thiazolo[5,4-*c*]quinoline which leads to them exhibiting their properties as important synthetic targets [5,6,7,8,9,10,11,12,13,14] due to their biological and pharmacological efficiency, such as DYRKIA inhibitors [15], HIV-1integrase inhibition [16], antimalarial [17], anticancer [18,19,20], anti-inflammatory [20,21], antituberculosis [22], antidepressant [23], anticonvulsant [24], antifungal [25], antihistamine [26], and antitumor [10,27,28,29] activity. In addition, these compounds play an important role by providing a new molecular framework for drug discovery [30]. Thus, thiazole and quinolone are privileged structures in the designing of a fused thiazoloquinazoline system, which is predictable to biodynamic properties with characteristic features. However, literature revealed a growing demand for the development of new syntheticprocesses includingpotential synthesisofactive substituted thiazole quinazoline systems like thiazolo[3,2-a]quinazoline, thiazolo[2,3-b]quinazoline, thiazolo[4,3-b]quinazoline, thiazolo[5,4-c]quinoline thiazolo[5,4-f]quinazoline, and thiazolo[4,5-h]quinazolin [31]. Moreover, after the extensive literature survey of the aforesaid, it was observed that there has been an increasing focus in the chemistry ofthiazolo[2,3-b]quinazoline because of the capability to exhibit an enormous variety of biological and pharmacological activities [32,33]. However, α,α dioxoketen dithioacetals are a pivotal starting material in synthetic chemistry due to their synthetic precursors that generate various heterocycle compounds [34,35]. The high potential ability of these intermediates to behave as polarized push-pull interacting systems on C–C double bonds makes them adaptable starting materials for the synthesis of a variety of novel heterocyclic compounds [36]. Based on the above discussion and in connection with our previous works on α,α dioxoketen dithioacetals as an efficient starting material [37,38], herein we report the novel convenient synthetic method for some new thiazolo[2,3-b]quinazolines **4**(**a**–**e**), pyrido[2′,3′:4,5]thiazolo[2,3-b]quinazolines **5**(**a**–**e**), **6**(**a**–**e**), and **7**(**a**–**e**), pyrano[2′,3′:4,5]thiazolo[2,3-b]quinazolines **8**(**a**–**e**), and benzo[4,5]thiazolo[2,3-b]quinazoline **9**(**a**–**e**) derivatives. Our efforts focused on the newer synthetic routes for these condensed compounds that are yet unreported. Inspired by our previous lab reports, we have evaluated the newly synthesized compounds as antibacterial and radical scavenging activities.

## 2. Results and Discussion

### 2.1. Chemistry

**Scheme 1:**
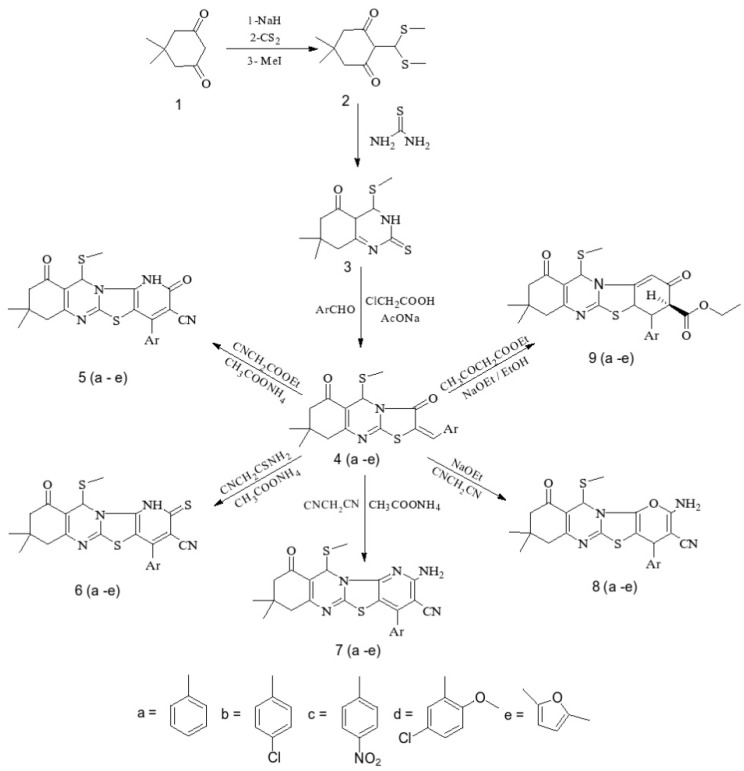
Synthesis of the target compounds **4** (**a**–**e**), **5** (**a**–**e**), **6** (**a**–**e**), **7** (**a**–**e**), **8** (**a**–**e**) and **9** (**a**–**e**).

The desired ketene-*S*,*S*-dithioacetal **2** was prepared in high yields by reacting the 5,5-dimethylcyclohexane-1,3-dione (dimedone) as an active methylene compound with carbon disulfide in the presence of sodium hydride as a base in dry benzene followed by alkylation’s with methyl iodide in a one-pot reaction [39] Scheme 1. Ketene-*S*,*S*-dithioacetal **2** possesses a replaceable active methylthio group -SCH_3_ which is activated by a carbonyl group. It is clear in these systems that there is a highly polarized push (dialkylthio)–pull (dicarbonyl) interaction on the C–C double bond, thus inducing the immediate reaction of ketene-*S*,*S*-dithioacetals **2** with thiourea in the presence of potassium bicarbonate as a catalyst in DMF under refluxing conditions to give the intermediate **3** in high yields as reported in the literature [40]. The reaction of compound **3** with aromatic aldehyde, chloroacetic acid, and anhydrous sodium acetate in an acetic acid-lactic anhydride mixture affords to the desired third key intermediate **4**(**a**–**e**) in high yields [41] as shown in Scheme 1.

The IR spectra of compound **3** displayed a broad peak at 3220–3375 cm^−1^ due to the-NH group and peaks at 1691 cm^−1^, 1647 cm^−1^, and 1245 cm^−1^ due to the carbonyl, -C=N, and -C=S groups, respectively. Its ^1^H-NMR spectrum showed a singlet signal at δ 2.44 ppm, corresponding to the -SCH_3_ group, and another singlet signal at δ 9.23 ppm corresponding to the -NH group. The ^13^C-NMR of compound **3** was featured by the signal at δ 18.42 ppm corresponding to the-SCH_3_ carbon.

The structures of compounds **4**(**a**–**e**) have been determined based on their CHN analysis, IR, ^1^H-NMR, and ^13^C-NMR data, which corroborated strongly to the structures assigned to the target molecules. The formation of compounds **4**(**a**–**e**) was ascertained by the manifestation of two carbonyl groups in the IR spectrum at 1667–1712 cm^−1^ along with an absorption band at 1632–1645 cm^−1^ due to -C=N group. Moreover, the ^1^H-NMR spectra exposed the disappearance of -NH signals of quinazoline moiety and the existence of a new singlet signal at δ 7.23 ppm assignable to -C=CH- and a multiplet at δ 7.42–7.52 assignable to aromatic protons of compound **4**(**a**) and doublets at δ 6.15–7.82 ppm assignable to aromatic protons of compound **4**(**b**–**e**). The ^13^C-NMR of compounds **4**(**a**–**e**) was characterized by two signals; one at δ 16.20–18.52 ppm corresponding to -SCH_3_ carbon and the other at δ 191.35–195.14 ppm corresponding to -C=O carbon.

**Scheme 2:**
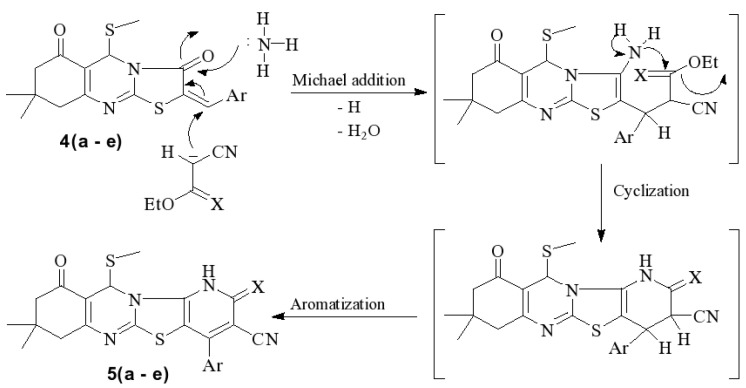
Synthesis of the compounds **5**(**a**–**e**).

The reaction of active intermediates **4**(**a**–**e**) with ethyl cyanoacetate, cyanothioacetamide, or malononitrile in a molar ratio 1:1 in the presence of ammonium acetate to give the products **5**(**a**–**e**), **6**(**a**–**e**), and **7**(**a**–**e**), respectively, through the Michael reaction, with the elimination ethyl alcohol and water is shown in general in Scheme 1. The structure of products **5**(**a**–**e**), **6**(**a**–**e**), and **7**(**a**–**e**) were proved on the basis of their CHN analysis and spectral data. The IR spectrum for products **5**(**a**–**e**) and **6**(**a**–**e**) showed a strong absorption band at 3318–3378 cm^−1^ due to the-NH group, whereas two absorption peaks at 4411–4450 and 3320–3379 cm^−1^ are featured for -NH_2_ group of compounds **7**(**a**–**e**). The IR spectra for compounds **5**(**a**–**e**), **6**(**a**–**e**), and **7**(**a**–**e**) also revealed absorption bands for -C=O, -C=S, and -CN groups at the expected regions. Moreover, the ^1^H-NMR spectra of compounds **5**(**a**–**e**), **6**(**a**–**e**), and **7**(**a**–**e**) revealed -NH signals and aromatic proton signals at the expected regions. The ^13^C-NMR spectra showed a signal at δ 16.33–18.68 ppm corresponding to-SCH_3_ and another signal at δ 191.31–193.87 ppm corresponding to -C=O carbon. All other carbon signals were observed at the expected regions. The proposed pathway for the preparation of this class of compounds is shown in Scheme 2. In this mechanism, the synthesized intermediate **4**(**a**–**e**) has an α-β unsaturated system and hence undergoes Michael addition with ethyl cyanoacetate attacking the β-carbon in the presence of ammonium acetate and results in a 1,4-addition intermediate. This intermediate then undergoes intramolecular cyclization very rapidly through nucleophilic acyl substitution to yield a six-membered ring which finally undergoes aromatization to give more stable heterocyclic products as compounds **5**(**a**–**e**) given in Scheme 2.

**Scheme 3:**
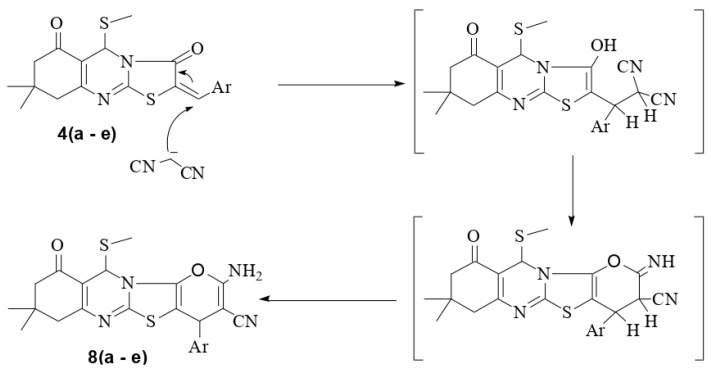
Synthesis of the compounds **8**(**a**–**e**).

Similarly, the reaction of compound **4**(**a**–**e**) with malononitrile with a molar ratio of 1:1 in the presence of sodium ethoxide solution leads to the product**8**(**a**–**e**) as shown in Scheme 1. The structures of products **8**(**a**–**e**) were determined according to their CHN analysis and spectral data. Their IR spectrum exhibited three different types of absorption. The first bands at 3437–3512 cm^−1^ and 3242–3340 cm^−1^ which is distinctive for -NH_2_ groups, while the second absorption band at 2211–2272 cm^−1^ is distinctive for -CN group, whereas the third absorption band at 1698–1747 cm^−1^ isdistinctive for -C=O groups. ^1^H-NMR spectra for compound **8**(**a**) as an example, displayed signals at δ 6.55 ppm assigned to the-NH_2_ group. Its ^13^C-NMR displayed signals at δ 42.11 ppm (Pyran C_4_), 118.43 (CN), 127.15, 127.46, 128.50, and 129.30 (aromatic carbons). The proposed pathways for the formation of these classes of products are illustrated in Scheme 3. In this mechanism, the synthesized intermediate **4**(**a**–**e**) undergoes Michael addition with malononitrile attacking the β-carbon in the presence of sodium ethoxide solution and results in a 1,4-addition product that swiftly undergoes intramolecular cyclization to finally give more stable heterocyclic aromatic products as compounds **8**(**a**–**e**) given in Scheme 3.

**Scheme 4:**
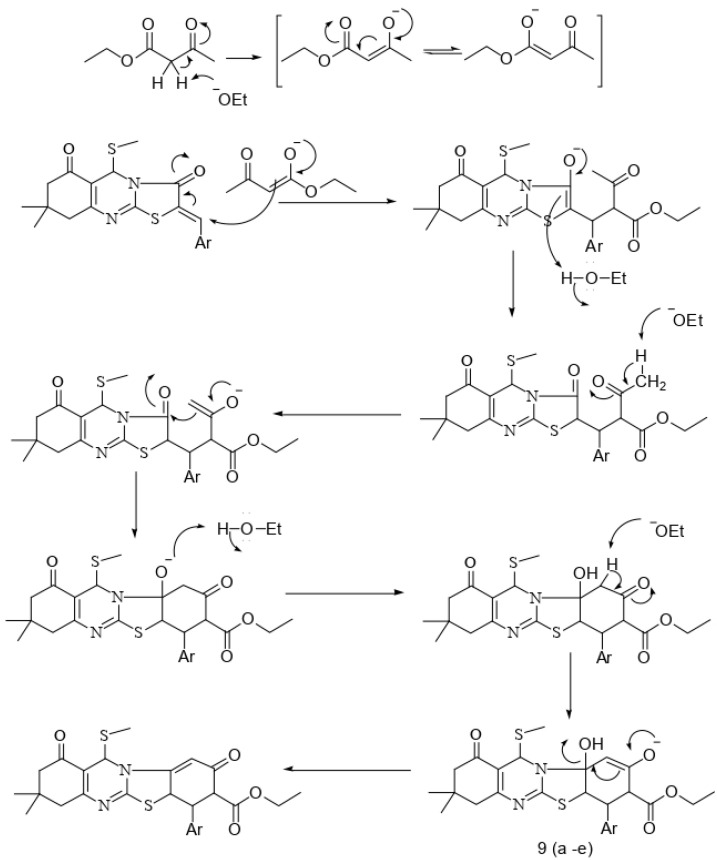
The mechanism pathway for the formation of compounds **9** (**a**–**e**).

Finally, the treatment of compound **4**(**a**–**e**) with ethyl acetoacetate in a molar ratio 1:1 in the presence of sodium ethoxide solution leads to the product **9**(**a**–**e**) as illustrated in Scheme 1. The compounds **9**(**a**–**e**) have been characterized by their CHN analysis and spectral data. The IR spectra for compounds**9**(**a**–**e**) also displayed three absorption bands at 1623–1695, 1685–1735, and 1726–1773 cm^−1^ for (3 C=O). Its ^1^H-NMR spectrum of **9**(**a**) as an example revealed in addition to the ethoxy group protons signals, a multiplet signal at δ 2.84 ppm, triplet signal at δ 3.16 ppm, and a singlet at δ 3.43 ppm, assigned to cyclohexene protons. The ^13^C-NMR spectrum of compound **9**(**a**) revealed signals at δ 16.11 ppm and 65.55 ppm corresponding to -CH_3_ and -OCH_2_. All other carbon signals were revealed at the expected regions. In this last series of compounds **9**(**a**–**e**), the mechanism follows the proposed pathway and goes via the Robinson annulation mechanism as illustrated in Scheme 4. The synthesized intermediates **4**(**a**–**e**) react with the ester, ethyl acetoacetate in the presence of sodium ethoxide solution and results in a 1,4-Michael addition intermediate followed by 1, 2- addition through intramolecular aldol condensation generating a six-membered ring. This then undergoes elimination finally giving Robinson annulation products as compounds **9**(**a**–**e**) depicted in Scheme 4.

### 2.2. Antibacterial Activity

Antibacterial activities of the synthesized compounds were tested by measuring the inhibition zone using the paper disc-diffusion method [42]. The data presented in Table 1 shows all synthesized compounds exhibit moderate to good activity against two or more bacterial strains. The synthesized compounds **5**(**b**), **6**(**b**), and **7**(**b**) showed vigorous activity against Gram-positive bacterial *B. subtilis* while the compounds **5**(**c**), **6**(**c**), **7**(**c**), and **8**(**c**) exhibit potent antibacterial activity against the Gram-negative bacterial strain *E. coli* and *P. aeruginosa* at a concentration of 100 μg/mL as illustrated in Table 1. However, among all the tested compounds, the compound **6**(**c**) showed maximum activity against *E. coli* and *P. aeruginosa* whereas the compounds **7**(**b**) and **8**(**c**) showed the maximum activity against *B. subtillis* and *S. aureus*, respectively. The tested compounds **6**(**b**), **7**(**a**), and **8**(**c**) exhibited good activity against the *E. coli* strain while the compounds **5**(**b**) and **8**(**b**) showed good activity against *B. subtilis*, as for the tested compounds **6**(**c**), **7**(**c**), and **8**(**c**) against *P. aeruginosa.* The results also revealed that some of the compounds tested in the series **4**(**a**–**e**) and **9**(**a**–**e**) showed the lowest inhibition while some of them showed no zone of inhibition against all the tested microorganisms as given in Table 1. However, the rest of the compounds showed low to moderate antibacterial activity against all the tested microorganisms at the same concentration. In terms of the structure–activity relationship, results suggest that the presence of pyrido or pyrano moieties bearing Oxo-, Thio-, or amino groups at position 2 and carbonitrile at position 3 along with substituted phenyl at position 4 might be the most relevant cause for enhancing the activity moreso, for those having strong electron-withdrawing nitro groups at the para position. Compound **6**(**c**) bearing Thio-, carbonitrile, and nitro groups at 2, 3, and 4 positions, respectively, displayed the maximum activity against *E. coli* and *P. aeruginosa*. However, the replacement of the thio group by the Oxo- group at position 2 did not affect the activity as much as the absence of a strong electron-withdrawing group at the para position of the phenyl moiety. Although, the presence of two electron–donating substituents simultaneously at ortho and meta positions as in compounds **5**(**d**), **6**(**d**), **7**(**d**), and **8**(**d**), resulted in low activity. Also, the replacement of the phenyl ring by a substituted fural group at the 4 position as in the compounds **5**(**e**), **6**(**e**), **7**(**e**), and **8**(**e**) might not favor the activity. Finally, the results indicate clearly that the absence of the pyrido and pyrano moieties such as in synthesized compounds **4**(**a**–**e**) or the presence of the benzo moiety bearing carboxylate group at the position 8 for instance in synthesized compounds **9**(**a**–**e**) might be the probable reason behind the reduced activity.

### 2.3. Antioxidant Activity

The 2,2-diphenyl-1-picryl-hydrazyl (DPPH) radical scavenging activity (RSA) evaluation is a standard assay in antioxidant activity studies and widely used as a rapid technique for assess the ability of compounds or extracts as scavengers of free radicals and hence evaluate the antioxidant activity of synthetics compounds [36,37,38]. In this work, the interaction of the synthesized compounds with stable DPPH free radical indicates their free radical scavenging ability. The majority of the synthesized compounds showed low to moderate interaction with the DPPH radical at 10 μg/mL concentration compared to vitamin C as standard. Maximum DPPH radical scavenging activity was observed in synthetic compounds **9**(**b**) and **9**(**c**) (*p < 0.05*), which possess a 4-Cl-phenyl and carboxylate group at position 7 and at position 8 of the condensed benzo[4,5]thiazolo[2,3-b]quinazoline moieties, respectively. The other interesting outcome was observed in synthetic compound **6**(**b**,**d**), **7**(**b**–**d**), **8**(**c**,**d**), and **9**(**a**,**d**,**e**) which displayed good radical scavenging activity compared to vitamin C at a 10 μg/mL concentration. The results obtained are depicted in Table 2. The data in Table 2 also revealed that the synthetic compounds **4**(**b**,**c**,**e**), **5**(**a**–**c**,**e**), **6**(**a**,**c**,**e**), and **8**(**a**,**b**,**e**) showed mild-to-moderate behavior as a radical scavenger compared to the standard vitamin C scavenging capacity, whereas the compounds **4**(**a**,**d**), **5**(**d**), and **7**(**a**,**e**) did not show any activity. The presence of benzo moiety bearing either or both a 4-electron-withdrawing-phenyl group and carboxylate group at the positions 7 and 8, respectively, mostly favor the activity particularly with a strong electron-withdrawing group such as NO_2_ which could be the most possiblereason behind the remarkable activity of the tested compound **9**(**c**).

## 3. Experimental Section

### 3.1. General Experimental Procedures

All chemicals used for the synthesis were purchased from the Padmashri scientific (Mysore, India) and Sigma-Aldrich (St. Louis, MO, USA) companies and used without further purification. Thomas Hoover melting point apparatus was used for the determination of melting points (°C, uncorrected). IR spectrum was recorded (KBr) with the help of a Shimadzu 8300 spectrometer, in the range 400–4000 cm^−1^. An elemental analysis was achieved on an Elementorvairo-EL instrument. ^1^H-NMR (400 MHz) and ^13^C-NMR (100 MHz) spectra were obtained with Sea 400 (Bruker, Middlesex County, MA, USA) utilizing CDCl_3_ as solvent and TMS as reference. δ ppm units were used to express chemical shifts.

### 3.2. Synthetic Procedures

#### General Procedure for the Synthesis of 7,7-dimethyl-4-(methylthio)-2-thioxo-2,3,7,8-tetrahydroquinazolin-5(6H)-one (**3**)

A mixture of α,α -dioxoketenedithioacetal **2** (1.2 g, 5 mmol), thiourea (0.38 g, 5 mmol), and anhydrous potassium carbonate (25 mg) in DMF was refluxed for 6 h. The progress of the reaction was spotted on TLC. After the completion of the reaction, the crude product was cooled to room temperature, diluted with water (3 × 10 mL), and then extracted to ethyl acetate (2 × 20 mL). The ethyl acetate layer was dried over Na_2_SO_4_, the solvent was removed, and the final product collected was purified by column chromatography utilizing chloroform/petroleum ether (3:1) as an eluent system to give pure solid compound 3 as orange crystals (1.03 g, 81.1%), m. p. 142–144 °C. The structure of compound 3 was established by IR, ^1^H and ^13^C-NMR, and CHN analysis as shown below.IR (KBr pellets cm^−1^)ν_max_,3375–3220 (NH), 1691 (C=O), 1647 (C=N), 1245 (C=S); ^1^H-NMR (400 MHz, CDCl_3_); δ 1.02 (s, 3H, -CH_3_); 1.25 (s,3H, -CH_3_); 2.18 (m, ^2^J = 18.55 Hz, 2H, -CH_A_H_B_); 2.43 (s, 3H, -SCH_3_);2.60 (s, 1H, -CH_2_-); 2.86 (s, 1H, -CH_2_-); 5.61 (s, 1H, -CH-); 9.83 (s, 1H, -NH); 11.05 (s, 1H, -NH);^13^C-NMR (100 MHz, CDCl_3_); δ 16.20, 28.10, 36.40, 41.24, 55.20, 92.34, 168.55, 183.49, 189.22, 197.62. Anal. calcd. for C_11_H_16_N_2_OS_2_: C, 51.53%; H, 6.29%, N, 10.93%. Found: C, 50.98%; H, 6.61%; N, 11.20%.

### 3.3. General Method for the Synthesis of Compounds **4**(**a**–**e**)

A mixture of 7,7-dimethyl-4-(methylthio)-2-thioxo-2,3,7,8-tetrahydroquinazolin-5(6H)-one 3 (1.27 g, 5 mmol), chloroacetic acid (0.47 g, 5 mmol), fused sodium acetate (1 g), and the appropriate aldehydes (5 mmol) in glacial acetic acid (30 mL) was refluxed for 6 h. The reaction mixture was poured into ice-cold water, the precipitate formed was filtered off and, after drying, purified by crystallization from the suitable solvent.

*(E)-2-benzylidene-8,8-dimethyl-5-(methylthio)-8,9-dihydro-2H-thiazolo[2,3-b]quinazoline-3,6(5H,7H)-dione***4**(**a**) was recrystallized from ethanol as a yellow crystalline solid (1.1 g, 57.3%), m. p.173–175 °C. IR (KBr pellets cm^−1^)ν_max_,1712, 1673 (2C=O), 1641 (C=N),1610 (C=C); ^1^H-NMR (400 MHz, CDCl_3_); δ 1.27 (s, 3H, -CH_3_); 1.38 (s, 3H, -CH_3_); 1.60 (s, 2H, -CH_2_-); 1.92 (s, 2H, -CH_2_-); 2.20 (s, 3H, -SCH_3_); 4.51 (s, 1H, -CH-); 7.42–7.52 (m, 5H, ArH); 7.61 (s, 1H, -C=CH-); ^13^C-NMR (100 MHz, CDCl_3_); δ 16.33, 25.11, 33.12, 37.51, 54.22, 56.10, 118.80, 128.16, 128.40, 128.94, 132.20, 138.12, 145.16, 152.29, 158.63, 168.33, 195.14. Anal. calcd. for C_20_H_20_N_2_O_2_S_2_: C, 62.47%; H, 5.24%, N, 7.29%. Found: C, 62.20%; H, 5.39%; N, 7.67%.

*(E)-2-(4-chlorobenzylidene)-8,8-dimethyl-5-(methylthio)-8,9-dihydro-2H-thiazolo[2,3-b]quinazoline-3,6(5H,7H)-dione***4**(**b**)was recrystallized from ethanol as orange crystalline solid(1.2 g, 80%), m. p. 152–154 °C. IR (KBr pellets cm^−1^)ν_max_,1698, 1675 (2C=O),1632 (C=N), 1616 (C=C); ^1^H-NMR (400 MHz, CDCl_3_); δ 1.18 (s, 3H, -CH_3_); 1.30 (s, 3H, -CH_3_); 1.74 (s, 2H, -CH_2_-); 1.98 (s, 2H, -CH_2_-); 2.33 (s, 3H, -SCH_3_); 4.84 (s, 1H, -CH-); 7.34 (d, *J* = 8.00 Hz, 2H, Ar); 7.53 (d, *J* = 8.00 Hz, 2H, Ar); 7.65 (s, 1H, -C=CH-); ^13^C-NMR (100 MHz, CDCl_3_); δ 17.20, 24.14, 32.52, 35.71, 54.10, 55.62, 115.73, 126.10, 127.80, 128.42, 130.90, 136.22, 141.55, 153.13, 155.10, 163.14, 191.33. Anal. calcd. for C_20_H_19_ClN_2_O_2_S_2_: C, 57.34%; H, 4.57%, N, 6.69%. Found: C, 57.48%; H, 4.49%; N, 6.81%.

*(E)-8,8-dimethyl-5-(methylthio)-2-(4-nitrobenzylidene)-8,9-dihydro-2H-thiazolo[2,3-b]quinazoline-3,6(5H,7H)-dione***4**(**c**)was recrystallized from dioxane as yellow crystalline solid (1.9 g, 88.4%), m. p.118–120 °C. IR (KBr pellets cm^−1^)ν_max_,1691, 1670 (2C=O), 1645 (C=N), 1611 (C=C);^1^H-NMR (400 MHz, CDCl_3_); δ 1.43 (s, 3H, -CH_3_); 1.50 (s, 3H, -CH_3_); 1.80 (s, 2H, -CH_2_-); 2.40 (s, 2H, -CH_2_-); 2.82 (s, 3H, -SCH_3_); 5.12 (s, 1H, -CH-); 7.55 (d, *J* = 8.40 Hz, 2H, Ar); 7.73 (d, *J* = 8.40Hz, 2H, Ar); 7.90 (s, 1H, -C=CH-); ^13^C-NMR (100 MHz, CDCl_3_); δ 15.44, 23.94, 33.42, 36.36, 54.70, 56.21, 116.80, 127.40, 128.23, 128.94, 132.10, 135.12, 144.62, 154.64, 156.11, 165.50, 194.53. Anal. calcd. for C_20_H_19_N_3_O_4_S_2_: C, 55.93%; H, 4.46%, N, 9.78%. Found: C, 55.48%; H, 4.70%; N, 9.43%.

*(E)-2-(5-chloro-2-methoxybenzylidene)-8,8-dimethyl-5-(methylthio)-8,9-dihydro-2H-thiazolo[2,3-b]quinazoline-3,6(5H,7H)-dione***4**(**d**) was recrystallized from chloroform/pet-ether as crystalline solid (1.3 g, 53.1%), m. p. 135–137 °C. IR (KBr pellets cm^−1^)ν_max_,1711, 1679 (2C=O), 1633 (C=N), 1600 (C=C);^1^H-NMR (400 MHz, CDCl_3_); δ 1.13 (s, 3H, -CH_3_); 1.20 (s, 3H, -CH_3_); 1.95 (s, 2H, -CH_2_-); 2.31 (s, 2H, -CH_2_-); 2.64 (s, 3H, -SCH_3_); 3.12 (s, 3H, -OCH_3_); 6.42 (s, 1H, -CH-); 7.25 (d, *J* = 8.20 Hz,1H, Ar);7.52 (s, 1H, Ar); 7.82 (d, *J* = 8.20 Hz,1H, Ar);7.96 (s, 1H, -C=CH-); ^13^C-NMR (100 MHz, CDCl_3_); δ 18.52, 24.34, 25.22, 32.62, 35.25, 52.15, 54.77, 115.22, 117.38, 118.66, 125.50, 126.73, 131.14, 132.55, 145.16, 155.22, 156.24, 158.30, 168.30, 191.68. Anal. calcd. for C_21_H_21_ClN_2_O_3_S_2_: C, 56.18%; H, 4.71%, N, 7.90%. Found: C, 56.67%; H, 4.40%; N, 7.21%.

*(E)-2-((2,5-dimethylfuran-3-yl)methylene)-8,8-dimethyl-5-(methylthio)-8,9-dihydro-2H-thiazolo[2,3-b]quinazoline-3,6(5H,7H)-dione***4**(**e**) was recrystallized from dioxane as yellow crystalline solid (1.4 g, 72.2%), m. p. 181–183 °C. IR (KBr pellets cm^−1^)ν_max_,1695, 1667 (2C=O), 1640 (C=N), 1632 (C=C);^1^H-NMR (400 MHz, CDCl_3_); δ 1.08(s, 3H, -CH_3_); 1.16 (s, 3H, -CH_3_); 1.92 (s, 2H, -CH_2_-); 2.20 (s, 2H, -CH_2_-); 2.45 (s, 3H, -CH_3_); 2.63 (s, 3H, -SCH_3_); 2.88 (s, 3H, -CH_3_); 5.82 (s, 1H, -CH-); 6.15 (d, J = 7.50 Hz,1H, Ar); 7.20 (d, J = 7.50 Hz,1H, Ar); 7.88 (s, 1H, -C=CH-); ^13^C-NMR (100 MHz, CDCl_3_); δ 16.20, 18.89, 23.52, 25.30, 30.12, 38.43, 51.43, 56.18, 112.13, 121.45, 123.70, 131.40, 142.75, 152.24, 156.15, 158.48, 160.60, 168.23, 194.56. Anal. calcd. for C_20_H_22_N_2_O_3_S_2_: C, 58.74%; H, 5.19%, N, 7.21%. Found: C, 58.38%; H, 5.60%; N, 7.52%.

### 3.4. Typical Procedure for the Synthesis of Compounds **5**(**a**–**e**)

A mixture of (E)-2-(4-chlorobenzylidene)-8,8-dimethyl-5-(methylthio)-8,9-dihydro-2H-thiazolo[2,3-b]quinazoline3,6(5H,7H)-dione **4**(**a**)(5 mmol), ethyl cyanoacetate (0.57 mL, 5 mmol) and ammonium acetate (0.077 g, 10 mmol) in n-butanol (30 mL) was refluxed for 4 h. After cooling, the precipitate formed was filtered off, dried, and recrystallized from the suitable solvent.

*8,8-dimethyl-11-(methylthio)-2,10-dioxo-4-phenyl-2,7,8,9,10,11-hexahydro-1H-pyrido[2’,3’:4,5]thiazolo[2,3-b]quinazoline-3-carbonitrile***5**(**a**) was recrystallized from ethanol as white crystalline solid (0.98 g, 43.8%), m. p.201–203 °C. IR (KBr pellets cm^−1^)ν_max_, 3340 (NH), 2219 (CN), 1775, 1691 (2C=O), 1655 (C=N); ^1^H-NMR (400 MHz, CDCl_3_); δ 1.12(s, 3H, -CH_3_); 1.24 (s, 3H, -CH_3_); 1.98 (s, 2H, -CH_2_-); 2.15 (s, 2H, -CH_2_-); 2.95 (s, 3H, -SCH_3_); 5.14 (s, 1H, -CH-); 7.12–7.48 (m,5H, Ar); 9.18 (s, 1H, -NH); ^13^C-NMR (100 MHz, CDCl_3_); δ 17.62, 25.33, 32.42, 37.63, 52.21, 58.66, 88.23, 114.30, 117.34, 126.15, 128.10, 128.45, 130.32, 134.57, 139.90, 155.75, 157.63, 162.38, 169.45, 196.14. Anal. calcd. for C_23_H_20_N_4_O_2_S_2_: C, 61.59%; H, 4.49%, N, 12.49%. Found: C, 61.40%; H, 4.16%; N, 11.98%.

*4-(4-chlorophenyl)-8,8-dimethyl-11-(methylthio)-2,10-dioxo-2,7,8,9,10,11-hexahydro-1H-pyrido[2’,3’:4,5]thiazolo[2,3-b]quinazoline-3-carbonitrile***5**(**b**) obtained from **4**(**b**). It was recrystallized from ethanol:benzene (2:1) as a white crystalline solid (1.2 g, 49.3%), m. p. 223–224 °C. IR (KBr pellets cm^−1^)ν_max_, 3355 (NH), 2268 (CN), 1743, 1687 (2C=O), 1645 (C=N); ^1^H-NMR (400 MHz, CDCl_3_); δ 1.08 (s, 3H, -CH_3_); 1.31 (s, 3H, -CH_3_); 1.99 (s, 2H, -CH_2_-); 2.27 (s, 2H, -CH_2_-); 2.98 (s, 3H, -SCH_3_); 5.38 (s, 1H, -CH-); 7.28 (d, J = 8.80 Hz, 2H, Ar); 7.82 (d, J = 8.80 Hz, 2H, Ar); 10.04 (s, 1H, -NH); ^13^C-NMR (100 MHz, CDCl_3_); δ 17.44, 26.16, 33.22, 40.11, 50.98, 58.13, 86.45, 115.67, 117.56, 126.78, 127.52, 128.45, 130.40, 131.34, 136.60, 155.15, 158.13, 162.24, 170.21, 197.54. Anal. calcd. for C_23_H_19_ClN_4_O_2_S_2_: C, 57.19%; H, 3.96%, N, 11.60%. Found: C, 57.79%; H, 3.52%; N, 11.11%.

*8,8-dimethyl-11-(methylthio)-4-(4-nitrophenyl)-2,10-dioxo-2,7,8,9,10,11-hexahydro-1H-pyrido[2’,3’:4,5]thiazolo[2,3-b]quinazoline-3-carbonitrile***5**(**c**) obtained from **4**(**c**). It was recrystallized from ethanol:benzene (2:1) as a yellow crystalline solid (1.8 g, 72.9%), m. p.197–198 °C. IR (KBr pellets cm^−1^)ν_max_, 3337 (NH), 2284 (CN), 1761, 1680 (2C=O), 1655 (C=N); ^1^H-NMR (400 MHz, CDCl_3_); δ 1.13 (s, 3H, -CH_3_); 1.26 (s, 3H, -CH_3_); 1.96 (s, 2H, -CH_2_-); 2.18 (s, 2H, -CH_2_-); 2.82 (s, 3H, -SCH_3_); 5.44 (s, 1H, -CH-); 7.30 (d, J = 8.42 Hz, 2H, Ar); 7.71 (d, J = 8.42 Hz, 2H, Ar); 10.98 (s, 1H, -NH); ^13^C-NMR (100 MHz, CDCl_3_); δ 16.56, 25.40, 32.56, 39.30, 51.46, 55.74, 87.12, 114.57, 116.62, 124.62, 129.45, 130.80, 131.54, 139.19, 146.78, 153.80, 156.25, 163.66, 170.33, 199.13. Anal. calcd. for C_23_H_19_N_5_O_4_S_2_: C, 55.97%; H, 3.88%, N, 14.19%. Found: C, 56.12%; H, 3.24%; N, 10.98%.

*4-(5-chloro-2-methoxyphenyl)-8,8-dimethyl-11-(methylthio)-2,10-dioxo-2,7,8,9,10,11-hexahydro-1H-pyrido[2’,3’:4,5]thiazolo[2,3-b]quinazoline-3-carbonitrile***5**(**d**) obtained from **4**(**d**). It was recrystallized from dilute ethanol as a colorless crystalline solid (1.7 g, 66.2%), m. p. 168–171 °C. IR (KBr pellets cm^−1^)ν_max_, 3370 (NH), 2265 (CN), 1758, 1692 (2CO), 1651 (C=N); ^1^H-NMR (400 MHz, CDCl_3_); δ 1.12(s, 3H, -CH_3_); 1.29 (s, 3H, -CH_3_); 1.92 (s, 2H, -CH_2_-); 2.08 (s, 2H, -CH_2_-); 2.80 (s, 3H, -SCH_3_); 3.41 (s, 3H, -OCH_3_), 4.98 (s, 1H, -CH-); 7.18 (d, J = 8.80 Hz, 1H, Ar); 7.44 (s, 1H, Ar); 7.68 (d, J = 8.80 Hz, 1H, Ar);9.38 (s, 1H, -NH); ^13^C-NMR (100 MHz, CDCl_3_); δ 18.78, 27.30, 33.23, 38.50, 51.14, 53.16, 54.66, 85.72, 114.62, 115.84, 115.98, 121.08, 125.42, 126.15, 130.12, 130.80, 136.28, 153.80, 155.70, 158.26, 160.90, 170.45, 193.46. Anal. calcd. for C_24_H_21_ClN_4_O_3_S_2_: C, 56.19%; H, 4.13%, N, 10.92%. Found: C, 56.25%; H, 4.78%; N, 11.34%.

*8,8-dimethyl-4-(5-methylfuran-2-yl)-11-(methylthio)-2,10-dioxo-2,7,8,9,10,11-hexahydro-1H-pyrido[2’,3’:4,5]thiazolo[2,3-b]quinazoline-3-carbonitrile***5**(**e**) obtained from **4**(**e**). It was recrystallized from dilute ethanol as a colorless crystalline solid (1.5 g, 66.4%), m. p. 189–191 °C. IR (KBr pellets cm^−1^)ν_max_, 3378 (NH), 2293 (CN), 1784, 1687 (2C=O), 1637 (C=N); ^1^H-NMR (400 MHz, CDCl_3_); δ 1.10(s, 3H, -CH_3_); 1.26 (s, 3H, -CH_3_); 1.90 (s, 2H, -CH_2_-); 2.23 (s, 2H, -CH_2_-); 2.45 (s, 3H, -SCH_3_); 2.80 (s, 3H, -CH_3_);4.91(s, 1H, -CH-); 7.45 (d, J = 8.00 Hz,1H, Ar); 7.60 (d, J = 8.00 Hz, 1H, Ar);9.24 (s, 1H, -NH); ^13^C-NMR (100 MHz, CDCl_3_); δ 11.32, 12.68, 15.36, 28.06, 32.98, 38.65, 50.56, 57.14, 87.12, 107.57, 116.64, 120.25, 121.26, 132.44, 136.71, 148.02, 154.14, 156.50, 157.78, 162.17, 170.23, 195.87. Anal. calcd. for C_23_H_22_N_4_O_3_S_2_: C, 58.39%; H, 4.45%, N, 12.38%. Found: C, 57.05%; H, 4.11%; N, 12.74%.

### 3.5. Typical Procedure for the Synthesis of Compounds **6**(**a**–**e**)

A mixture of (*E*)-2-(4-chlorobenzylidene)-8,8-dimethyl-5-(methylthio)-8,9-dihydro-2H-thiazolo[2,3-b]quinazoline3,6(5H,7H)-dione **4**(**a**)(5 mmol), cyanothioacetamide (0.5 g, 5 mmol) and ammonium acetate (0.077 g, 10 mmol) in 30 mL n-butanol was refluxed for 4 h. The precipitate formed was filtered off and, after drying, purified by crystallization from the suitable solvent.

*8,8-dimethyl-11-(methylthio)-10-oxo-4-phenyl-2-thioxo-2,7,8,9,10,11-hexahydro-1H-pyrido[2’,3’:4,5]thiazolo[2,3-b]quinazoline-3-carbonitrile***6**(**a**) was recrystallized from ethanol as red crystalline solid (1.2 g, 51.7%). m. p. 148–150 °C. IR (KBr pellets cm^−1^)ν_max_, 3311 (NH), 2270 (CN), 1730(C=O),1648 (C=N), 1250 (C=S); ^1^H-NMR (400 MHz, CDCl_3_); δ 1.04 (s, 3H, -CH_3_); 1.15 (s, 3H, -CH_3_); 2.08 (s, 2H, -CH_2_-); 2.40 (s, 2H, -CH_2_-); 2.84 (s, 3H, -SCH_3_); 4.75 (s, 1H, -CH-); 7.10–7.55 (m, 5H, Ar); 9.08 (s, 1H, -NH); ^13^C-NMR (100 MHz, CDCl_3_); δ 17.32, 26.17, 31.52, 40.23, 50.92, 57.12, 82.44, 102.20, 113.54, 128.20, 128.98, 129.64, 130.62, 132.17, 151.73, 153.12, 159.56, 163.48, 170.75, 191.13. Anal. calcd. for C_22_H_20_N_4_OS_3_: C, 59.46%; H, 4.34%, N, 12.06%. Found: C, 59.94%; H, 4.78%; N, 12.55%.

*4-(4-chlorophenyl)-8,8-dimethyl-11-(methylthio)-10-oxo-2-thioxo-2,7,8,9,10,11-hexahydro-1H-pyrido[2’,3’:4,5]thiazolo[2,3-b]quinazoline-3-carbonitrile***6**(**b**) obtained from **4**(**b**). It was recrystallized from ethanol as an orange crystalline solid (2.1 g, 84%). m. p. 160–162 °C. IR (KBr pellets cm^−1^)ν_max_, 3325 (NH), 2297 (CN), 1760, (C=O),1653 (C=N), 1248 (C=S); ^1^H-NMR (400 MHz, CDCl_3_); δ 1.14 (s, 3H, -CH_3_); 1.38 (s, 3H, -CH_3_); 1.70 (s, 2H, -CH_2_-); 2.40 (s, 2H, -CH_2_-); 2.84 (s, 3H, -SCH_3_); 4.93 (s, 1H, -CH-); 7.11 (d, J = 8.80 Hz, 2H, Ar); 7.62 (d, J = 8.80 Hz, 2H, Ar); 9.25 (s, 1H, -NH); ^13^C-NMR (100 MHz, CDCl_3_); δ 16.64, 25.82, 31.02, 41.55, 52.35, 55.40, 83.20, 102.88, 114.16, 125.51, 127.80, 128.17, 129.60, 132.18, 154.25, 156.40, 160.21, 164.22, 173.51, 193.88. Anal. calcd. for C_23_H_19_ClN_4_OS_3_: C, 55.35%; H, 3.84%, N, 11.23%. Found: C, 56.91%; H, 3.34%; N, 11.11%.

*8,8-dimethyl-11-(methylthio)-4-(4-nitrophenyl)-10-oxo-2-thioxo-2,7,8,9,10,11-hexahydro-1H-pyrido[2’,3’:4,5]thiazolo[2,3-b]quinazoline-3-carbonitrile***6**(**c**) obtained from **4**(**c**). It was recrystallized from ethanol:benzene (1:1) as an orange crystalline solid (2.1 g, 82.4%). m. p. 204–206 °C. IR (KBr pellets cm^−1^)ν_max_, 3344 (NH), 2234 (CN), 1715, (C=O),1627 (C=N),1253 (C=S); ^1^H-NMR (400 MHz, CDCl_3_); δ 1.11 (s, 3H, -CH_3_); 1.26 (s, 3H, -CH_3_); 1.80 (s, 2H, -CH_2_-); 2.30 (s, 2H, -CH_2_-); 2.88 (s, 3H, -SCH_3_); 5.12 (s, 1H, -CH-); 7.18 (d, J = 8.80 Hz, 2H, Ar); 7.60 (d, J = 8.80 Hz, 2H, Ar); 9.44 (s, 1H, -NH); ^13^C-NMR (100 MHz, CDCl_3_); δ 17.50, 25.02, 32.30, 40.71, 52.23, 58.33, 84.13, 107.21, 117.65, 121.41, 130.80, 131.28, 132.50, 139.43, 151.35, 155.23, 155.83, 160.11, 174.51, 194.19. Anal. calcd. for C_23_H_19_N_5_O_3_S_3_: C, 54.21%; H, 3.76%, N, 13.74%. Found: C, 55.05%; H, 3.50%; N, 13.32%.

*4-(5-chloro-2-methoxyphenyl)-8,8-dimethyl-11-(methylthio)-10-oxo-2-thioxo-2,7,8,9,10,11-hexahydro-1H-pyrido[2’,3’:4,5]thiazolo[2,3-b]quinazoline-3-carbonitrile***6**(**d**) obtained from **4**(**d**). It was recrystallized from ethanol:benzene (1:2) as yellow crystalline solid (2.3 g, 86.8%). m. p. 193–195 °C. IR (KBr pellets cm^−1^)ν_max_, 3354 (NH), 2228 (CN), 1726, (C=O),1650 (C=N), 1231 (C=S); ^1^H-NMR (400 MHz, CDCl_3_); δ 1.17 (s, 3H, -CH_3_); 1.35 (s, 3H, -CH_3_); 1.82 (s, 2H, -CH_2_-); 2.10 (s, 2H, -CH_2_-); 2.78 (s, 3H, -SCH_3_); 3.24 (s, 3H, -OCH_3_), 4.93(s, 1H, -CH-); 7.23 (d, J = 8.40 Hz,1H, Ar); 7.56 (s, 1H, Ar); 7.85 (d, J = 8.40 Hz,1H, Ar); 9.89 (s, 1H, -NH); ^13^C-NMR (100 MHz, CDCl_3_); δ 18.68, 27.78, 30.43, 39.36, 50.84, 56.65, 58.12, 81.44, 104.33, 113.23, 115.58, 120.24, 126.51, 128.45, 130.25, 131.79, 150.21, 153.30, 155.35, 158.98, 163.12, 173.55, 196.16. Anal. calcd. for C_24_H_21_ClN_4_O_2_S_3_: C, 54.48%; H, 4.00%, N, 10.59%. Found: C, 54.80%; H, 3.56%; N, 10.10%.

*8,8-dimethyl-4-(5-methylfuran-2-yl)-11-(methylthio)-10-oxo-2-thioxo-2,7,8,9,10,11-hexahydro-1H-pyrido[2’,3’:4,5]thiazolo[2,3-b]quinazoline-3-carbonitrile***6**(**e**) obtained from **4**(**e**). It was recrystallized from dilute ethanol as yellow crystalline solid (1.3 g, 55.6%). m. p. 156–158 °C. IR (KBr pellets cm^−1^)ν_max_, 3318 (NH), 2183 (CN), 1711, (C=O), 1638 (C=N), 1245 (C=S); ^1^H-NMR (400 MHz, CDCl_3_); δ 1.07 (s, 3H, -CH_3_); 1.19 (s, 3H, -CH_3_); 1.83 (s, 2H, -CH_2_-); 2.28 (s, 2H, -CH_2_-); 2.54 (s, 3H, -SCH_3_); 2.86 (s, 3H, -CH_3_);5.43(s, 1H, -CH-); 7.36 (d, J = 7.50 Hz,1H, Ar); 7.67 (d, J = 7.50 Hz, 1H, Ar); 10.14 (s, 1H, -NH); ^13^C-NMR (100 MHz, CDCl_3_); δ 10.86, 12.32, 14.18, 25.66, 30.25, 39.15, 52.41, 58.23, 89.78, 110.62, 112.62, 114.54, 116.36, 131.38, 148.62, 151.14, 153.76, 157.51, 159.43, 160.45, 170.44, 197.46. Anal. calcd. for C_23_H_22_N_4_O_2_S_3_: C, 56.39%; H, 4.30%, N, 11.96%. Found: C, 55.98%; H, 4.83%; N, 12.14%.

### 3.6. Typical Procedure for the Synthesis of Compounds **7**(**a**–**e**)

A solution of (*E*)-2-(4-chlorobenzylidene)-8,8-dimethyl-5-(methylthio)-8,9-dihydro-2H-thiazolo[2,3-b]quinazoline3,6(5H,7H)-dione **4**(**a**) (5 mmol), and malononitrile (0.34 g, 5 mmol) in the presence of ammonium acetate (0.077 g, 10 mmol) in 30 mL n-butanol was refluxed for 4 h. After cooling, the precipitate formed was filtered off and, after drying, purified by crystallization from the suitable solvent.

*2-amino-8,8-dimethyl-11-(methylthio)-10-oxo-4-phenyl-8,9,10,11-tetrahydro-7H-pyrido[2’,3’:4,5]thiazolo[2,3-b]quinazoline-3-carbonitrile***7**(**a**) was recrystallized from ethanol:benzene (1:1) as a colorless crystalline solid (1.9 g, 84.4%), m. p. 155–157 °C. IR (KBr pellets cm^−1^)ν_max_, 3435, 3320 (NH_2_), 2234 (CN), 1726(C=O), 1632, 1610 (2C=N); ^1^H-NMR (400 MHz, CDCl_3_); δ 1.11(s, 3H, -CH_3_); 1.43 (s, 3H, -CH_3_); 2.13 (s, 2H, -CH_2_-); 2.46 (s, 2H, -CH_2_-); 2.83 (s, 3H, -SCH_3_); 5.44 (s, 1H, -CH-); 6.73 (s, 2H, -NH_2_); 7.36–7.76 (m,5H, Ar); ^13^C-NMR (100 MHz, CDCl_3_); δ 18.51, 25.45, 31.87, 41.35, 52.33, 64.23, 87.61, 114.22, 121.65, 127.36, 127.80, 129.18, 131.48, 139.13, 153.54, 154.12, 157,65, 158.13, 168.28, 196.32. Anal. calcd. for C_23_H_21_N_5_OS_2_: C, 61.72%; H, 4.73%, N, 15.56%. Found: C, 61.55%; H, 4.12%; N, 16.17%.

*2-amino-4-(4-chlorophenyl)-8,8-dimethyl-11-(methylthio)-10-oxo-8,9,10,11-tetrahydro-7H-pyrido[2’,3’:4,5]thiazolo[2,3-b]quinazoline-3-carbonitrile***7**(**b**) obtained from **4**(**b**). It was recrystallized from ethanol:benzene (1:1) as a pale yellow crystalline solid (1.8 g, 75%), m. p. 112–114 °C. IR (KBr pellets cm^−1^)ν_max_, 3415, 3345 (NH_2_), 2256 (CN), 1740(C=O), 1641, 1616 (2C=N); ^1^H-NMR (400 MHz, CDCl_3_); δ 1.09(s, 3H, -CH_3_); 1.52 (s, 3H, -CH_3_); 2.20 (s, 2H, -CH_2_-); 2.65 (s, 2H, -CH_2_-); 2.93 (s, 3H, -SCH_3_); 5.12 (s, 1H, -CH-); 7.04 (s, 2H, -NH_2_); 7.48 (d, J = 8.80 Hz, 2H, Ar); 7.96 (d, J = 8.80 Hz, 2H, Ar); ^13^C-NMR (100 MHz, CDCl_3_); δ 18.21, 25.84, 30.45, 40.12, 50.72, 65.68, 84.11, 112.95, 124.16, 127.50, 127.80, 128.24, 131.56, 133.56, 151.65, 153.04, 158.05, 158.68, 167.77, 197.33. Anal. calcd. for C_23_H_20_ClN_5_OS_2_: C, 57.31%; H, 4.18%, N, 14.53%. Found: C, 56.91%; H, 4.43%; N, 14.85%.

*2-amino-8,8-dimethyl-11-(methylthio)-4-(4-nitrophenyl)-10-oxo-8,9,10,11-tetrahydro-7H-pyrido[2’,3’:4,5]thiazolo[2,3-b]quinazoline-3-carbonitrile***7**(**c**) obtained from **4**(**c**). It was recrystallized from ethanol:benzene (1:2) as a yellow crystalline solid (0.9 g, 36.6%), m. p. 141–143 °C. IR (KBr pellets cm^−1^)ν_max_, 3396, 3324 (NH_2_), 2210 (CN), 1755(C=O), 1654, 1610 (2C=N); ^1^H-NMR (400 MHz, CDCl_3_); δ 1.17(s, 3H, -CH_3_); 1.48 (s, 3H, -CH_3_); 2.28 (s, 2H, -CH_2_-); 2.75 (s, 2H, -CH_2_-); 2.88 (s, 3H, -SCH_3_); 5.23 (s, 1H, -CH-); 6.98 (s, 2H, -NH_2_); 7.40 (d, J = 8.80 Hz, 2H, Ar); 7.82 (d, J = 8.80 Hz, 2H, Ar); ^13^C-NMR (100 MHz, CDCl_3_); δ 16.81, 24.92, 30.22, 40.52, 51.66, 63.45, 83.61, 114.32, 124.95, 128.44, 128.80, 130.14, 143.16, 147.20, 152.64, 154.60, 158,01, 158.80, 169.73, 191.31. Anal. calcd. for C_23_H_20_N_6_O_3_S_2_: C, 56.08%; H, 4.09%, N, 17.06%. Found: C, 55.78%; H, 3.89%; N, 17.46%.

*2-amino-4-(5-chloro-2-methoxyphenyl)-8,8-dimethyl-11-(methylthio)-10-oxo-8,9,10,11-tetrahydro-7H-pyrido[2’,3’:4,5]thiazolo[2,3-b]quinazoline-3-carbonitrile***7**(**d**) obtained from **4**(**d**). It was recrystallized from ethanol as a white crystalline solid (2.2 g, 85.9%), m. p. 167–169 °C. IR (KBr pellets cm^−1^)ν_max_, 3411, 3352 (NH_2_), 2270 (CN), 1741(C=O), 1637, 1616 (2C=N); ^1^H-NMR (400 MHz, CDCl_3_); δ 1.06(s, 3H, -CH_3_); 1.20 (s, 3H, -CH_3_); 2.23 (s, 2H, -CH_2_-); 2.64 (s, 2H, -CH_2_-); 2.80 (s, 3H, -SCH_3_); 3.16 (s, 3H, -OCH_3_), 4.94 (s, 1H, -CH-); 6.87 (s, 2H, -NH_2_); 7.20 (d, J = 8.20 Hz,1H, Ar); 7.49 (s, 1H, Ar); 7.92 (d, J = 8.20 Hz,1H, Ar); ^13^C-NMR (100 MHz, CDCl_3_); δ 16.33, 24.20, 29.82, 40.13, 48.46, 52.12, 64.43, 84.71, 114.55, 116.76, 123.20, 127.13, 127.60, 128.36, 128.74, 130.80, 132.65, 153.06, 154.16, 155,61, 158.24, 158,80, 168.08, 195.94. Anal. calcd. for C_24_H_22_ClN_5_O_2_S_2_: C, 56.30%; H, 4.33%, N, 13.68%. Found: C, 56.40%; H, 4.74%; N, 13.11%.

*2-amino-8,8-dimethyl-4-(5-methylfuran-2-yl)-11-(methylthio)-10-oxo-8,9,10,11-tetrahydro-7H-pyrido[2’,3’:4,5]thiazolo[2,3-b]quinazoline-3-carbonitrile***7**(**e**) obtained from **4**(**e**). It was recrystallized from ethanol:hexane(2:1) as a white crystalline solid (1.1 g, 48.7%), m. p. 167–169 °C. IR (KBr pellets cm^−1^)ν_max_, 3450, 3379 (NH_2_), 2258 (CN), 1758(C=O), 1637, 1620 (2C=N); ^1^H-NMR (400 MHz, CDCl_3_); δ 1.14(s, 3H, -CH_3_); 1.26 (s, 3H, -CH_3_); 2.33 (s, 2H, -CH_2_-); 2.60 (s, 2H, -CH_2_-); 2.79 (s, 3H, -SCH_3_); 2.90, (s, 3H, CH_3_), 4.94 (s, 1H, -CH-); 6.28 (s, 2H, -NH_2_); 6.68 (d, J = 7.50 Hz,1H, Ar); 7.42 (d, J = 7.50 Hz,1H, Ar); ^13^C-NMR (100 MHz, CDCl_3_); δ 11.45, 12.63, 14.10, 26.80, 32.12, 40.56, 50.55, 64.41, 82.51, 104.60, 112.50, 123.18, 125.60, 131.33, 146.12, 152.34, 152,89, 156.13, 158,27, 158.53, 158.91, 193.87. Anal. calcd. for C_23_H_23_N_5_O_2_S_2_: C, 58.52%; H, 4.69%, N, 15.51%. Found: C, 58.90%; H, 4.13%; N, 15.81%.

### 3.7. Typical Procedure for the Synthesis of Compounds **8**(**a**–**e**)

A mixture of (*E*)-2-benzylidene-8,8-dimethyl-5-(methylthio)-8,9-dihydro-2H-thiazolo[2,3-b]quinazoline-3,6(5H,7H)-dione **4**(**a**) (1.27 g, 5 mmol), and malononitrile (0.34 g, 5 mmol) in sodium ethoxide solution (5%, 15 mL) was refluxed for 6 h. Then the reaction mixture was cooled, poured onto ice/cold water, and acidified by diluted HCl to pH = 5.5–6. The precipitate formed was filtered off and, after drying, purified by crystallization from the suitable solvent.

*2-amino-8,8-dimethyl-11-(methylthio)-10-oxo-4-phenyl-4,7,8,9,10,11-hexahydropyrano[2’,3’:4,5]thiazolo[2,3-b]quinazoline-3-carbonitrile***8**(**a**) was recrystallized from ethanol:hexane (1:1) as colorless crystalline solid (1.5 g, 66.7%), m. p. 185–187 °C. IR (KBr pellets cm^−1^)ν_max_, 3464, 3290 (NH_2_), 2250 (CN), 1747(CO), 1646 (C=N); ^1^H-NMR (400 MHz, CDCl_3_); δ 1.02(s, 3H, -CH_3_); 1.28 (s, 3H, -CH_3_); 2.07 (s, 2H, -CH_2_-); 2.36 (s, 2H, -CH_2_-); 2.81 (s, 3H, -SCH_3_); 3.67 (s, 1H, -CH); 4.32 (s, 1H, -CH); 6.55 (s, 2H, -NH_2_); 7.42–7.80 (m, 5H, Ar); ^13^C-NMR (100 MHz, CDCl_3_); δ 18.33, 26.68, 31.51, 39.78, 41.91, 51.63, 54.87, 60.36, 73.43, 117.50, 127.15, 127.46, 129.30, 130.52, 143.07, 148.72, 153.27, 157.33, 159,17, 195.69. Anal. calcd. for C_23_H_22_N_4_O_2_S_2_: C, 61.31%; H, 4.92%, N, 12.43%. Found: C, 61.98%; H, 4.51%; N, 12.87%.

*2-amino-4-(4-chlorophenyl)-8,8-dimethyl-11-(methylthio)-10-oxo-4,7,8,9,10,11-hexahydropyrano[2’,3’:4,5]thiazolo[2,3-b]quinazoline-3-carbonitrile***8**(**b**) obtained from **4**(**b**). It was recrystallized from ethanol as a white crystalline solid (1.4 g, 57.6%), m. p. 114–116 °C. IR (KBr pellets cm^−1^)ν_max_, 3437, 3245 (NH_2_), 2211 (CN), 1720(CO), 1633 (C=N); ^1^H-NMR (400 MHz, CDCl_3_); δ 1.17(s, 3H, -CH_3_); 1.44 (s, 3H, -CH_3_); 2.20 (s, 2H, -CH_2_-); 2.58 (s, 2H, -CH_2_-); 2.84 (s, 3H, -SCH_3_); 3.55 (s, 1H, -CH); 4.34 (s, 1H, -CH); 6.49 (s, 2H, -NH_2_); 7.36 (d, J = 8.20 Hz, 2H, Ar); 7.87 (d, J = 8.20 Hz, 2H, Ar); ^13^C-NMR (100 MHz, CDCl_3_); δ 18.27, 26.43, 33.16, 38.88, 39.25, 50.34, 56.21, 58.25, 71.65, 120.12,128.50, 128.60, 129.96, 131.66, 141.62, 148.08, 152.46, 157.11, 158,78, 195.11. Anal. calcd. for C_23_H_21_ClN_4_O_2_S_2_: C, 56.96%; H, 4.36%, N, 11.55%. Found: C, 57.10%; H, 4.20%; N, 11.47%.

*2-amino-8,8-dimethyl-11-(methylthio)-4-(4-nitrophenyl)-10-oxo-4,7,8,9,10,11-hexahydropyrano[2’,3’:4,5]thiazolo[2,3-b]quinazoline-3-carbonitrile***8**(**c**) obtained from **4**(**c**). It was recrystallized from ethanol:benzene (1:2) as a white crystalline solid (1.9 g, 76%), m. p. 128–130 °C. IR (KBr pellets cm^−1^)ν_max_, 3512, 3320 (NH_2_), 2242 (CN), 1716(CO), 1643 (C=N); ^1^H-NMR (400 MHz, CDCl_3_); δ 1.10(s, 3H, -CH_3_); 1.40 (s, 3H, -CH_3_); 2.28 (s, 2H, -CH_2_-); 2.60 (s, 2H, -CH_2_-); 2.89 (s, 3H, -SCH_3_); 3.61(s, 1H, -CH); 4.52 (s, 1H, -CH); 6.79 (s, 2H, -NH_2_); 7.28 (d, J = 8.20 Hz, 2H, Ar); 7.80 (d, J = 8.20 Hz, 2H, Ar); ^13^C-NMR (100 MHz, CDCl_3_); δ 17.11, 26.21, 33.04, 36.64, 38.72, 50.45, 55.12, 58.30, 71.57, 121.22, 124.14, 125.83, 130.50, 145.16, 146.51, 149.08, 153.56, 158.92, 159.98, 197.42. Anal. calcd. for C_23_H_21_N_5_O_4_S_2_: C, 55.74%; H, 4.27%, N, 14.13%. Found: C, 55.23%; H, 4.80%; N, 13.95%.

*2-amino-4-(5-chloro-2-methoxyphenyl)-8,8-dimethyl-11-(methylthio)-10-oxo-4,7,8,9,10,11-hexahydropyrano[2’,3’:4,5]thiazolo[2,3-b]quinazoline-3-carbonitrile***8**(**d**) obtained from **4**(**d**). It was recrystallized from ethanol:benzene (1:2) as a white crystalline solid (1.2 g, 46.2%), m. p. 153–154 °C. IR (KBr pellets cm^−1^)ν_max_, 3467, 3340 (NH_2_), 2272 (CN), 1730(CO), 1640 (C=N); ^1^H-NMR (400 MHz, CDCl_3_); δ 1.13(s, 3H, -CH_3_); 1.37 (s, 3H, -CH_3_); 2.34 (s, 2H, -CH_2_-); 2.55 (s, 2H, -CH_2_-); 2.80 (s, 3H, -SCH_3_); 3.15(s, 3H, -OCH_3_); 3.85(s, 1H, -CH); 4.60 (s, 1H, -CH); 6.98 (s, 2H, -NH_2_); 7.32 (d, J = 8.08 Hz,1H, Ar); 7.50 (s, 1H, Ar); 7.98 (d, J = 8.08 Hz,1H, Ar); ^13^C-NMR (100 MHz, CDCl_3_); δ 17.19, 26.13, 30.82, 32.78, 38.23, 50.66, 55.24, 57.41, 60.02, 72.13, 111.35, 119.83, 120.94, 127.50, 128.32, 130.25, 132.14, 149.85, 156.30, 158.12, 159.42, 160.32, 199.12. Anal. calcd. for C_24_H_23_ClN_4_O_3_S_2_: C, 55.97%; H, 4.50%, N, 10.88%. Found: C, 55.09%; H, 4.72%; N, 10.15%.

*2-amino-8,8-dimethyl-4-(5-methylfuran-2-yl)-11-(methylthio)-10-oxo-4,7,8,9,10,11-hexahydropyrano[2’,3’:4,5]thiazolo[2,3-b]quinazoline-3-carbonitrile***8**(**e**) obtained from **4**(**e**). It was recrystallized from ethanol as a yellow crystalline solid (1.2 g, 46.2%), m. p. 117–119 °C. IR (KBr pellets cm^−1^)ν_max_, 3447, 3260 (NH_2_), 2222 (CN), 1698 (CO), 1639 (C=N); ^1^H-NMR (400 MHz, CDCl_3_); δ 1.19 (s, 3H, -CH_3_); 1.24 (s, 3H, -CH_3_); 1.90 (s, 2H, -CH_2_-); 2.25 (s, 2H, -CH_2_-); 2.64 (s, 3H, -SCH_3_); 2.84 (s, 3H, -CH_3_); 3.61 (s, 1H, -CH); 4.23 (s, 1H, -CH); 6.98 (s, 2H, -NH_2_); 7.12 (d, J = 9.80.10 Hz,1H, Ar); 7.23 (d, J = 9.80 Hz, 1H, Ar); ^13^C-NMR (100 MHz, CDCl_3_); δ 11.20, 13.53, 14.35, 25.92, 31.50, 32.65, 39.48, 50.37, 55.53, 58.21, 71.91, 107.83, 108.61, 113.36, 131.10, 147.66, 149.15, 149.80, 153.44, 158.70, 160.35, 193.65. Anal. calcd. for C_23_H_24_N_4_O_3_S_2_: C, 58.13%; H, 4.88%, N, 12.33%. Found: C, 58.76%; H, 4.23%; N, 12.02%.

### 3.8. Typical Procedure for the Synthesis of Compounds **9**(**a**–**e**)

A mixture of (*E*)-2-benzylidene-8,8-dimethyl-5-(methylthio)-8,9-dihydro-2H-thiazolo[2,3-b]quinazoline-3,6(5H,7H)-dione **4**(**a**)(1.27 g, 5 mmol), and ethyl acetoacetate (0.77 mL, 6 mmol) in sodium ethoxide solution (5%, 25 mL) was refluxed for 4 h. Then the reaction mixture was evaporated under reduced pressure, the residue formed was filtered off and, after drying, purified by crystallization from the suitable solvent.

*(8S)-ethyl 3,3-dimethyl-12-(methylthio)-1,9-dioxo-7-phenyl-2,3,4,6a,7,8,9,12-octahydro-1H-benzo[4,5]thiazolo[2,3-b]quinazoline-8-carboxylate***9**(**a**) was recrystallized from ethanol:petrolum ether (2:1) as a green crystalline solid (2 g, 80.7%), m. p. 183–185 °C. IR (KBr pellets cm^−1^)ν_max_, 1726, 1714, 1695 (3CO), 1630 (C=N); ^1^H-NMR (400 MHz, CDCl_3_); δ 1.07 (s, 3H, -CH_3_); 1.18 (s, 3H, -CH_3_); 1.47 (t, 3H, J = 7.10 Hz, -CH_3_); 1.74 (s, 2H, -CH_2_-); 1.92 (s, 2H, -CH_2_-); 2.16 (s, 3H, -SCH_3_); 2.84 (m, 1H, -CH-cyclohexene); 3.16 (m, 1H, -CH-cyclohexene); 3.43 (m,1H, -CH-cyclohexene); 4.25 (q, 2H, J = 7.00 Hz, -OCH_2_); 4.36 (s, 1H, -CH); 6.60 (s, 1H, =CH); 7.13–7.93 (m, 5H, Ar); ^13^C-NMR (100 MHz CDCl_3_); δ 12.23, 16.11, 26.58, 26.91, 30.40, 39.47, 43.20, 53.47, 58.25, 59.48, 65.55, 100.08, 124.67, 128.16, 128.47, 132.32, 147.14, 152.64, 158.66, 161,20, 170.30, 192.58, 198.44. Anal. calcd. for C_26_H_28_N_2_O_4_S_2_: C, 62.88%; H, 5.68%, N, 5.64%. Found: C, 62.13%; H, 5.92%; N, 6.08%.

*(8S)-ethyl 7-(4-chlorophenyl)-3,3-dimethyl-12-(methylthio)-1,9-dioxo-2,3,4,6a,7,8,9,12-octahydro-1H-benzo[4,5]thiazolo[2,3-b]quinazoline-8-carboxylate***9**(**b**) obtained from **4**(**b**). It was recrystallized from ethanol:petroleum ether (2:1) as a pale green crystalline solid (2.4 g, 90.2%), m. p. 163–165 °C. IR (KBr pellets cm^−1^)ν_max_, 1755, 1712, 1678 (3CO), 1641 (C=N); ^1^H-NMR (400 MHz, CDCl_3_); δ 1.03 (s, 3H, -CH_3_); 1.20 (s, 3H, -CH_3_); 1.44 (t, 3H, J = 7.10 Hz,-CH_3_); 1.80 (s, 2H, -CH_2_-); 1.95 (s, 2H, -CH_2_-); 2.20 (s, 3H, -SCH_3_); 2.72 (m, 1H, -CH-cyclohexene); 3.13 (m, 1H, -CH-cyclohexene); 3.40 (m,1H, -CH-cyclohexene); 4.16 (q, 2H, J = 7.00 Hz, -OCH_2_); 4.38 (s, 1H, -CH); 6.55 (s, 1H, =CH); 7.30 (d, J = 8.20 Hz, 2H, Ar); 7.65 (d, J = 8.20 Hz, 2H, Ar);^13^C-NMR (100 MHz CDCl_3_); δ 17.00, 18.31, 24.36, 24.83, 33.20, 38.14, 45.11, 52.07, 56.60, 58.18, 62.15, 115.63, 125.33, 127.11, 133.45, 135.17, 144.13, 152.54, 160.25, 164.25, 169.16, 193.11, 197.45. Anal. calcd. for C_26_H_27_ClN_2_O_4_S_2_: C, 58.80%; H, 5.12%, N, 5.27%. Found: C, 58.04%; H, 4.92%; N, 5.54%.

*(8S)-ethyl 3,3-dimethyl-12-(methylthio)-7-(4-nitrophenyl)-1,9-dioxo-2,3,4,6a,7,8,9,12-octahydro-1H-benzo[4,5]thiazolo[2,3-b]quinazoline-8-carboxylate***9**(**c**) obtained from **4**(**c**). It was recrystallized from ethanol as a yellow crystalline solid (1.8 g, 66.4%), m. p. 214–216 °C. IR (KBr pellets cm^−1^)ν_max_, 1736, 1685, 1623 (3CO), 1652 (C=N); ^1^H-NMR (400 MHz, CDCl_3_); δ 1.09 (s, 3H, -CH_3_); 1.23 (s, 3H, -CH_3_); 1.50 (t, 3H, J = 7.10 Hz, -CH_3_); 1.72 (s, 2H, -CH_2_-); 1.90 (s, 2H, -CH_2_-); 2.24 (s, 3H, -SCH_3_); 2.72 (m, H, -CH-cyclohexene); 3.19 (m, 1H, -CH-cyclohexene); 3.48 (m,1H, -CH-cyclohexene); 4.30 (q, 2H, J = 7.00 Hz, -OCH_2_); 4.48 (s, 1H, -CH); 6.35 (s, 1H, =CH); 7.27 (d, J = 8.20 Hz, 2H, Ar); 7.50 (d, J = 8.20 Hz, 2H, Ar); ^13^C-NMR (100 MHz CDCl_3_); δ 17.74, 18.84, 24.91, 25.52, 32.66, 38.11, 46.22, 54.30, 56.46, 58.32, 62.25, 103.23, 124.72, 127.55, 132.17, 147.60, 155,43, 156.30, 160.42, 165.11, 169.60, 191.38, 196.82. Anal. calcd. for C_26_H_27_N_3_O_6_S_2_: C, 57.65%; H, 5.02%, N, 7.76%. Found: C, 57.34%; H, 4.66%; N, 7.11%.

*(8S)-ethyl 7-(5-chloro-2-methoxyphenyl)-3,3-dimethyl-12-(methylthio)-1,9-dioxo-2,3,4,6a,7,8,9,12-octahydro-1H-benzo[4,5]thiazolo[2,3-b]quinazoline-8-carboxylate***9**(**d**) obtained from **4**(**d**). It was recrystallized from ethanol as a pale green crystalline solid (1.4 g, 49.8%), m. p. 221–223 °C. IR (KBr pellets cm^−1^)ν_max_, 1773, 1735, 1670 (3CO), 1658 (C=N); ^1^H-NMR (400 MHz, CDCl_3_); δ 1.14 (s, 3H, -CH_3_); 1.29 (s, 3H, -CH_3_); 1.52 (t, 3H, J = 7.10 Hz, -CH_3_); 1.78 (s, 2H, -CH_2_-); 1.94 (s, 2H, -CH_2_-); 2.14 (s, 3H, -SCH_3_); 2.60 (m, 1H, -CH-cyclohexene); 3.28 (m, 1H, -CH-cyclohexene); 3.40 (s, 3H, -OCH_3_); 3.51 (m,1H, -CH-cyclohexene); 4.16 (q, 2H, J = 7.00 Hz, -OCH_2_); 4.40 (s, 1H, -CH); 6.46 (s, 1H, =CH); 7.30 (d, J = 8.08 Hz,1H, Ar); 7.56 (s, 1H, Ar); 7.90 (d, J = 8.08 Hz,1H, Ar); ^13^C-NMR (100 MHz, CDCl_3_); δ 16.52, 18.24, 22.30, 24.85, 32.47, 37.18, 43.68, 53.44, 55.18, 58.42, 58.60, 62.35, 103.87, 114.56, 125.80, 127.78, 131.45, 132.06, 137.83, 152.45, 156.21, 159.38, 164.72, 167.15, 194.18, 195.62. Anal. calcd. for C_27_H_29_ClN_2_O_5_S_2_: C, 57.79%; H, 5.21%, N, 4.99%. Found: C, 57.20%; H, 5.03%; N, 5.42%.

*(8S)-ethyl 7-(2,5-dimethylfuran-3-yl)-3,3-dimethyl-12-(methylthio)-1,9-dioxo-2,3,4,6a,7,8,9,12-octahydro-1H-benzo[4,5]thiazolo[2,3-b]quinazoline-8-carboxylate***9**(**e**) obtained from **4**(**e**). It was recrystallized from ethanol:hexane (1:3) as a pale yellow crystalline solid (1.9 g, 76%), m. p. 172–174 °C. IR (KBr pellets cm^−1^)ν_max_, 1760, 1730, 1681 (3CO), 1638 (C=N); ^1^H-NMR (400 MHz, CDCl_3_); δ 1.10 (s, 3H, -CH_3_); 1.19 (s, 3H, -CH_3_); 1.42 (t, 3H, J = 7.10 Hz, -CH_3_); 1.65 (s, 2H, -CH_2_-); 1.90 (s, 2H, -CH_2_-); 2.18 (s, 3H, -SCH_3_); 2.34 (s, 3H, -OCH_3_); 2.60 (m, H, -CH-cyclohexene); 3.18 (m, 1H, -CH-cyclohexene); 3.90(s, 3H, -OCH_3_); 4.19 (m,1H, -CH-cyclohexene); 4.30 (q, 2H, J = 7.00 Hz,-OCH_2_); 4.53 (s, 1H, -CH); 6.70 (s, 1H, =CH); 7.41 (d, J = 10.80.10 Hz,1H, Ar); 7.68 (d, J = 10.80 Hz, 1H, Ar);^13^C-NMR (100 MHz, CDCl_3_); δ 12.24, 15.30, 17,24, 18.63, 21.60, 26.11, 33.47, 40.25, 45.51, 52.23, 56.20, 58.11, 61.57, 106.92, 111.44, 127.19, 130.45, 148.23, 151.80, 159.43, 162.19, 163.89, 168.86, 193.22, 198.14. Anal. calcd. for C_26_H_30_N_2_O_5_S_2_: C, 59.98%; H, 5.64%, N, 5.60%. Found: C, 59.36%; H, 5.80%; N, 5.38%.

### 3.9. Biological Activity

#### 3.9.1. Antibacterial Activity

The antibacterial efficacy of the synthesized compounds was evaluated by measuring the inhibition zone using the paper disc-diffusion method. The experimental results were expressed as the mean ± standard deviation (n = 3). Group comparisons were performed using one-way ANOVA followed by Tukey’s post hoc test. A *p*-value of 0.05 was considered statistically significant. In the process, two Gram-positive bacteria (*Bacillus subtilis NCIM 2063 and Staphylococcus aureus NCIM 2079*) and two Gram-negative bacteria (*Escherichia coli NCIM 2065* and *Pseudomonas aeruginosa NCIM 5029*) were used to evaluate the antibacterial activities. The medium was prepared from molten nutrient and Mueller Hinton agar. Ciprofloxacin was used as the standard antibiotic while the discs without extracts loaded with organic solvents were used as the negative control. The four bacterial strains were tested with a 100 μg/mL concentration. Each compound was dissolved in DMSO at a concentration of 100 μg/mL, 6 mm diameter Whatman filter paper discs were soaked with 1 mL solution of the 100 μg/mL concentration for each compound, and then these saturated paper discs were inoculated at the center of each Petri dish a having bacterial lawn in triplicate. The plates were then incubated at 37 °C for 48 h, and the inhibition zone that appeared around the paper disc in each plate was determined by measuring its diameter. The results are illustrated in Table 1 [43].

#### 3.9.2. DPPH Radical Scavenging Activity

Antioxidant activity of compounds was determined using DPPH^•^ as described by Blois [44,45,46]. All the synthetic compounds were taken at a concentration of 10 µg/mL and mixed with 5 mL of 0.1 mM methanolic solution of DPPH^•^ and incubated at 20 °C for 20 min in darkness. The control was prepared as above without a compound, and methanol was used for the baseline correction. Changes in the absorbance of the samples were measured at 517 nm using a UV–visible spectrophotometer (Shimadzu 160A). All the tests were performed in duplicates. RSA was expressed as percentage activity using the formula:

RSA (%) = [(A_0_ − A_1_/A_0_ × 100)]

where A_0_ is the measurement of DPPH solution without compound and A_1_ is the measurement of DPPH solution with compound. The RSA of ascorbic acid was also measured and compared with all synthesized compounds.

## 4. Conclusions

In summary, the formation of new thiazolo[2,3-b]quinazolines **4**(**a**–**e**), pyrido[2′,3′:4,5]thiazolo[2,3-b]quinazolines {**5**(**a**–**e**), **6**(**a**–**e**), and **7**(**a**–**e**)}, pyrano[2′,3′:4,5]thiazolo[2,3-b]quinazolines **8**(**a**–**e**), and benzo[4,5]thiazolo[2,3-b]quinazoloine was achievedusing the starting material 3 which was synthesized from ketene-*S*,*S*-acetals 2. All the obtained compounds were characterized by elemental analysis, IR, ^1^H-NMR, and ^13^C-NMR. The synthesized compounds were screened for their antibacterial and antioxidant activity. In general, the tested compounds **5**(**b**), **6**(**b**), and **7**(**b**) showed high activity against Gram-positive bacterial *B. subtilis* more than Gram-negative bacteria, while the compounds **5**(**c**), **6**(**c**), **7**(**c**), and **8**(**c**) exhibited potent antibacterial activity against the Gram-negative bacterial strains *E. coli* and *P. aeruginosa*. The compounds **6**(**c**), **7**(**b**), and **8**(**c**) with the presence of pyrido or pyrano moieties bearing Oxo-, Thio-, or amino groups at position 2 and carbonitrile at position 3, and substituted phenyl at position 2 or 4 were most active against *E. coli*, *B. subtilis,* and *P. aeruginosa,* respectively. The results also revealed that the other displayed good to moderate along with low or no inhibition. In the case of antioxidant activity, the compounds **9**(**b**) and **9**(**c**) exhibited effective radical scavenging activity (RSA), while synthetic compounds **6**(**b**,**d**), **7**(**b**–**d**), **8**(**c**,**d**), and **9**(**a**,**d**,**e**)have shown good activity. The results also revealed that the other compounds showed moderate, low, or no antioxidant activity.

## Figures and Tables

**Table 1:** Antibacterial activity of the synthesized compounds.

Compounds	Bacterial Strains			
	*E. coli*	*B. subtilis*	*S. aureus*	*P. aeruginosa*
**4a**	NA	NA	NA	NA
**4b**	NA	6 ± 0.66	NA	4 ± 1.50
**4c**	2 ± 1.35	NA	4 ± 1.03	3 ± 1.23
**4d**	3 ± 0.29	NA	3 ± 1.24	4 ± 1.52
**4e**	NA	NA	2 ± 1.35	NA
**5a**	11 ± 1.08	10 ± 0.30	11 ± 1.04	9 ± 1.03
**5b**	19.± 0.61	18 ± 1.55	13 ± 1.76	6 ± 1.46
**5c**	26 ± 0.26	12 ± 0.48	10 ± 0.80	16 ± 1.61
**5d**	15 ± 0.40	14 ± 1.20	14 ± 1.07	11 ± 1.20
**5e**	11 ± 1.33	14 ± 1.39	12 ± 1.36	10 ± 1.03
**6a**	18 ± 1.24	9 ± 1.09	10 ± 1.23	9 ± 1.32
**6b**	22 ± 0.50	15 ± 1.05	14 ± 1.54	10 ± 1.55
**6c**	29 ± 1.08	13 ± 1.03	11 ± 1.10	18 ± 1.20
**6d**	18 ± 1.60	9 ± 1.42	10 ± 1.56	10 ± 1.46
**6e**	15 ± 0.18	7 ± 0.87	10 ± 1.21	7 ± 1.11
**7a**	22 ± 0.18	9 ± 1.40	15 ± 1.60	9 ± 1.40
**7b**	13 ± 0.33	19 ± 0.64	9 ± 1.43	9 ± 1.83
**7c**	28 ± 1.06	10 ± 1.61	14 ± 1.12	16 ± 1.42
**7d**	17 ± 0.91	13 ± 1.05	16 ± 1.15	10 ± 1.45
**7e**	11 ± 1.50	7 ± 1.10	13 ± 1.30	9 ± 1.20
**8a**	18 ± 1.41	14 ± 1.44	12 ± 1.61	8 ± 1.36
**8b**	12 ± 1.31	15 ± 0.37	10 ± 1.81	10 ± 1.21
**8c**	22 ± 1.20	11 ± 1.20	19 ± 1.09	17 ± 1.60
**8d**	18 ± 0.13	7 ± 1.13	18 ± 1.13	9 ± 1.53
**8e**	14 ± 1.51	8 ± 1.75	13 ± 1.42	7 ± 1.52
**9a**	NA	8 ± 1.52	NA	NA
**9b**	5 ± 1.29	4 ± 1.35	4 ± 1.40	3 ± 1.70
**9c**	4 ± 1.35	2 ± 0.40	4 ± 1.25	3 ± 1.66
**9d**	NA	3 ± 1.36	NA	NA
**9e**	NA	5 ± 1.17	NA	NA
**Ciprofloxacin**	31 ± 0.82	20 ± 1.28	28 ± 1.14	19 ± 0.92

Zone of inhibition in mm. Gram-positive bacterial strains: *S. aureus*—*Staphylococcus aureus*; *B. subtillis*—*Bacillus subtillis*. Gram-negative bacterialstrains: *E. coli*—*Escherichia coli*; *P. aeruginosa*—*Pseudomonas aeruginosa*. The concentration of test compounds was 100 μg/mL. Solvent which used DMSO. NA = not active. ND = not determined. The data represent mean value (SEM).

**Table 2:** Antioxidant activity of the synthesized novel series of the synthesized compounds.

Compounds	DPPH Radical Scavenging Activity (%) at 10 μg/mL
**4a**	NA
**4b**	38 ± 0.62
**4c**	57 ± 1.45
**4d**	NA
**4e**	58 ± 1.38
**5a**	30 ± 1.18
**5b**	46.± 0.21
**5c**	57 ± 0.56
**5d**	NA
**5e**	49 ± 1.13
**6a**	68 ± 1.24
**6b**	81 ± 0.71
**6c**	67 ± 1.58
**6d**	74 ± 1.26
**6e**	60 ± 0.88
**7a**	ND
**7b**	70 ± 1.63
**7c**	74 ± 1.56
**7d**	77 ± 0.81
**7e**	ND
**8a**	60 ± 1.31
**8b**	68 ± 1.14
**8c**	73 ± 1.84
**8d**	71 ± 0.63
**8e**	69 ± 1.43
**9a**	73 ± 1.13
**9b**	88 ± 1.63
**9c**	90 ± 1.15
**9d**	80 ± 1.87
**9e**	84 ± 1.63
**Vitamin C**	94.8 ± 0.55

Antioxidant activities were expressed in percentages compared with standard Vitamin C and E, respectively. NA = not active. ND = not determined. The data represent the mean value (SEM) of three duplicates.

## Data Availability

The data presented in this study are available on request from the corresponding author.

## Sample Availability

Not available.
